# Alcohol Use Disorders (AUD) among Tuberculosis Patients: A Study from Chennai, South India

**DOI:** 10.1371/journal.pone.0019485

**Published:** 2011-05-17

**Authors:** Mohanarani Suhadev, Beena E. Thomas, Raja Sakthivel M, Murugesan P, Chandrasekaran V, Niruparani Charles, Durga R, Auxilia M, Trini A. Mathew, Fraser Wares

**Affiliations:** 1 Tuberculosis Research Centre, Chetput, Chennai, Tamilnadu, India; 2 Office of the WHO Representative to India, New Delhi, India; 3 Division of Infectitious Diseases, University of Mississipi Medical Center, Jackson, Mississippi, United States of America; Fundació Institut Germans Trias i Pujol; Universitat Autònoma de Barcelona CibeRES, Spain

## Abstract

**Background:**

Alcohol Use Disorders (AUDs) among tuberculosis (TB) patients are associated with nonadherence and poor treatment outcomes. Studies from Tuberculosis Research Centre (TRC), Chennai have reported that alcoholism has been one of the major reasons for default and mortality in under the DOTS programme in South India. Hence, it is planned to conduct a study to estimate prevalence of alcohol use and AUDs among TB patients attending the corporation health centres in Chennai, India.

**Methodology:**

This is a cross-sectional cohort study covering 10 corporation zones at Chennai and it included situational assessment followed by screening of TB patients by a WHO developed Alcohol Use Disorders Identification Test AUDIT scale. Four zones were randomly selected and all TB patients treated during July to September 2009 were screened with AUDIT scale for alcohol consumption.

**Results:**

Out of 490 patients, 66% were males, 66% were 35 years and above, 57% were married, 58% were from the low monthly income group of <Rs 5000 per month. No females reported alcohol use. Overall, out of 490 TB pts, 29% (141) were found to consume alcohol. Among 141 current drinkers 52% (73) had an AUDIT score of >8. Age (>35 years), education (less educated), income (<Rs 5000 per month), marital status (separated/divorced) and treatment category (Category 2) were statistically significant for TB patients with alcohol use than those TB patients without alcohol use.

**Conclusions:**

AUD among TB patients needs to be addressed urgently and the findings suggest the importance of integrating alcohol treatment into TB care.

## Introduction

Tuberculosis (TB) remains a major global public health problem [1]. TB is one of the most important problems in India, with 1.98 million new cases per year, comprising over 20% of the global totalfor incident cases [2]. The Revised National Tuberculosis Control Programme (RNTCP) of India is the second largest programme in the world. India's Revised National Tuberculosis Control Programme (RNTCP), an adoption of the internationally recommended Directly Observed Treatment Short course (DOTS) strategy, focuses on providing free quality sputum smear microscopy for diagnosis as well as quality drugs for treatment free of cost. This strategy also provides decentralized treatment services close to patients' residence under direct observation with the help of government health workers and community volunteers [3].

Alcohol abuse is implicated in a wide variety of diseases, disorders and injuries, as well as many social and legal problems [4]–[6]. It has long been evident that there is an association between alcohol abuse and risk of tuberculosis (TB). A recent systematic review by Lonnrot et al [7] showed that the risk of active TB is substantially elevated in people who drink more than 40 g alcohol per day, and/or have an alcohol use disorder (AUD). This may be due to both increased risk of infection related to specific social mixing patterns associated with alcohol use, as well as influence on the immune system of alcohol itself and of alcohol related conditions In addition, alcohol disorders not only place individuals at increased risk for acquiring a number of diseases, but once people acquire a disease like TB, alcohol places them at higher risk for poor outcome and death [8]. A review of Tuberculosis Research Centre studies over a decade reveals that longer delay in TB diagnosis has also been associated with alcoholism (30 vs. 15 days; p<0.01) [9]. In south India, it has also been found that excessive alcohol intake is one of the major risk factors for treatment non-compliance and mortality under the Government of India's DOTS. [10], [11].

It is generally that in many settings health care providers do not screen for alcohol use disorders and its associated impact on the prognosis while treating major illnesses, including in patients suffering with TB. As part of the Integrated Management of Physician – delivered Alcohol Care for Tuberculosis (IMPACT) trial, Greenfield and her colleagues have developed a multidisciplinary model to manage AUDS among TB patients in Tomsk, Russia and the Alcohol Use Disorders Identification Test (AUDIT) was incorporated into routine assessment of all patients starting TB treatment [12]. In India, there is a dearth of information on prevalence of alcohol use and AUD amongst TB patients and its impact on adherence and disease progression. Detecting alcohol use disorders, specifically alcohol abuse and dependence, provides a critical opportunity for early intervention efforts to reduce adverse impacts of consumption [13]. It is against this background that the reported study was carried out. The findings from this study will assist the national TB programme in India to develop effective intervention strategies for TB patients with problems related to alcohol use. The aim of the study is to estimate prevalence of recent alcohol use among TB patients attending the Corporation clinics. and to estimate the prevalence of AUD amongst TB patients who consume alcohol, their treatment outcomes, and to explore the challenges in treatment management from the perspective of health providers and patients.

## Methods

**Figure 1:**
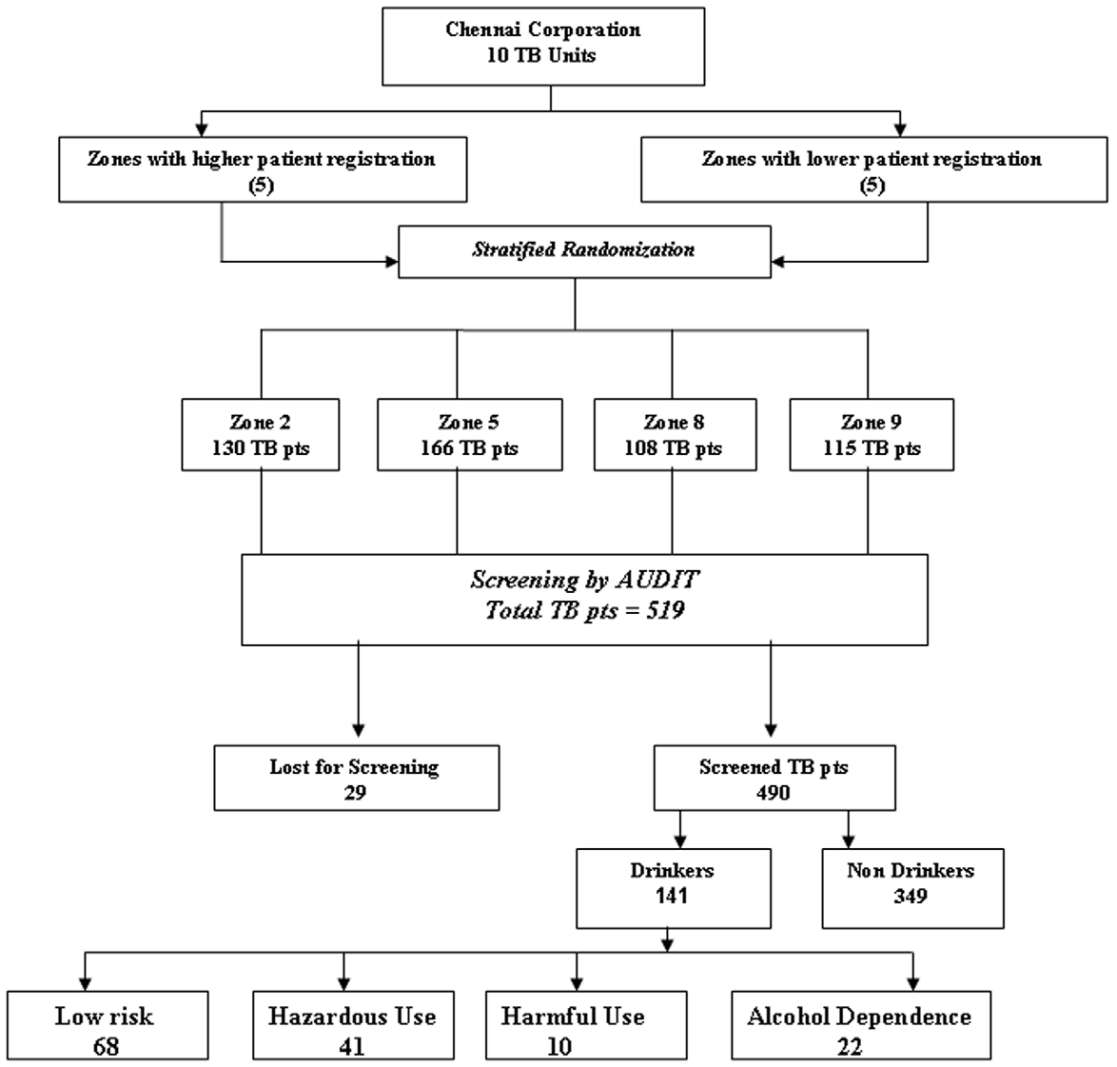
Research Design of the study.

This was a cross sectional cohort study covering 10 Tuberculosis Units (TU) in Chennai Corporation, South India (Figure 1). The overall study was conducted in phases and included both quantitative and qualitative methods. This paper will focus on the quantitative data collected firstly via a situational assessment and then via screening of TB patients registered under the Revised National TB Control Programme (RNTCP).

### 1. Situational assessment

The first phase of the study was a situational analysis to study the structure of the RNTCP programme in the Corporation zones in order to estimate the number of registered TB patients in one quarter, prevalence of alcohol use, reasons for default, experiences and perceptions of the patients as well as health providers on the need for alcohol intervention in the TB control programme. This was done via an examination of treatment records of the Corporation of Chennai's health department and discussions with key informants which included health providers, patients and their families. We referred to the documents released by the Government of India's Central TB Division for the organisation of the RNTCP in India in general and in Chennai in particular^2^.

### 2. Screening of TB patients using the AUDIT scale in randomly selected zones

The study population includes all new TB patients registered for TB treatment in the selected 4 zones in Chennai Corporation. Quarterly reports for the randomized 4 zones were obtained from the RNTCP centre. There were totally 519 TB patients started on TB treatment under DOTS from the 4 zones during July to September 2009. Out of 519 TB patients 29 patients were lost for AUDIT screening due to various reasons (Death, out of station, long default, migration etc;) and the remaining 490 TB patients were screened after getting their consent.

Screening of patients was done to estimate prevalence of alcohol use and AUDs defined as alcohol abuse or dependence. A simple screening tool – the Alcohol Use Disorders Identification Test (AUDIT-WHO 2001) was introduced to screen for hazardous alcohol use and AUDs among TB patients [14], [15]. The AUDIT has been internationally validated on primary health care patients [16] and used previously in Goa, India with industrial workers and primary care samples [17]–[20]. The scale was administered by trained counselors in the treatment centers.

Data was cross checked for correctness and the collected data was analyzed by SPSS (version-12). Results were calculated as frequencies (%) and Charts and graphs were used to explain the research design and the distribution of AUDs. A comparison was made between the drinkers and non-drinkers among TB patients for baseline demographic characteristics to find out the association between patient characteristics and alcohol use . The Chi square test was used to identify independent variables for alcohol use.

### Ethics Statement

The study protocol and instruments, including the informed consent forms, were approved by the Scientific Advisory Committee and the Institutional Review Board of the TB Research Centre and written informed consent obtained from all the study participants.

### Description of study sites

This study was carried out in Chennai which is the capital of Taminadu from South India. In Tamilnadu, prohibition is not in force and taxes from alcohol sales constitute a larger proportion of state income. Since 1983, Tamilnadu State Marketing Corporation (TASMAC) is the monopoly liquor seller in the state and alcohol served in units of millilitres or “millies” the term used by patrons [21], [22]. The most popular beverages are whisky, rum, brandy and beer. All these products are available in bottles of 750 ml. Alcoholic beverages in wine shops are either by the full bottle or by units of 45 ml. Alcohol content in Indian-manufactured foreign liquor (IMFL) used to be 42.8%. Here, drinks are consumed without water, soda or other additives. Our measures of alcohol involvement were not standardized along the lines of the AUDIT. Standard drink is an unfamiliar concept in India and detailed information on beverage specific drink sizes in India has not yet been documented. According to National Survey of Drug Abuse, 2004 [23], prevalence of alcohol use in adult men in Chennai ranges from 16.7% to 34.4%. Besides, the age of initiation to alcohol is going down and the young are being lured towards alcohol use.

The study area is urban Chennai where TB investigations and treatment are offered through the RNTCP programmes keeping patient's reach and care as its prime concern. For administrative purposes, the city is divided into 10 Corporation Zones and 155 Divisions. Patients diagnosed with tuberculosis are given DOTS in accordance with the RNTCP policies. All TB patients are treated with Short Course Chemotherapy (SCC) regimens in 3 different categories. Based on the Nature/severity of the disease and the Patients' exposure to previous anti-tubercular treatments, RNTCP classifies tuberculosis patients in to three Treatment Categories that are similar to the WHO TB patient's diagnostic Categories.

## Results

### Situational Analysis data

There are 10 corporation zones (Tuberculosis Units) under Chennai Corporation and each zone has got 3 to 7 TB microscopy and treatment centres. There are a total of 43 microscopy centres including private hospitals in Chennai Corporation where the RNTCP has been implemented. Standardized short-course chemotherapy is provided to new patients from a DOT centre close to the patients' house. Each zone covers a population of roughly 500,000. The baseline data on total number of patients on different regimens cure rates, treatment defaulter rates, reasons for default and the number of patients who consume alcohol were obtained from the study sites.

According to RNTCP records for the year 2008, zone 3 (Pulianthope TU) had maximum number of new TB patients on DOTS whereas zone 7 (Thanthai Periyar Dispensary) had minimum number of TB patients on DOTS for one year. The records pointed out to alcoholism being one of the reasons for default for TB treatment. However it was also found that there was no proper screening in place to measure alcoholism. This was recorded based on the visits made by the health visitors in the event of patient defaulting for treatment . This was also based on self reports of patients or a family member. However the general opinion among the health providers was that patients and families attending TB clinics required proper information on alcohol abuse and the potential adverse effects of alcohol on TB The need for alcohol intervention programes among TB patients who were dependant on alcohol was strongly expressed.

### Screening of TB patients for alcohol use using AUDIT

Based on the findings from the situational analysis, stratified random sampling was used to identify the zones from where patients were to be recruited for screening for alcohol use using AUDIT. Two zones were selected randomly from these 2 groups of high and low number of registered TB patients. Zones 2 and 5 were selected from the zones with the high number of TB patients and zones 8 and 9 from those with the low number of TB patients were selected for inclusion in the prospective phase of the study. All TB patients from these 4 zones who were enrolled for treatment under RNTCP from July to September 2009 were screened using the AUDIT questionnaire.

The screening and interview was done by trained counselors after obtaining their informed consent from the patients. A brief semi-structured interview schedule was used to elicit socio-demographic information which was followed by the administration of the AUDIT questionnaire. Those patients who consumed alcohol and who had an AUDIT score of >8 or more are recommended as indicators of hazardous and harmful alcohol use as well as possible alcohol dependence. AUDIT questions were translated into the local language Tamil before administration and it was administered by Tamil speaking interviewers who are well trained in getting correct responses from the study participants. Privacy and confidentiality were ensured for all study participants. AUDIT screening was not done as a part of any general health assessment.

### Socio demographic profile of all TB patients registered in the quarter July to September in 4 zones (Table 1)

**Table 1:** Baseline characteristics of total TB patients screened (n = 490).

Characteristics	Total patients	
	No	%
Male	322	168
Female	34	66
No formal education	76	16
School educated	360	73
College educated	54	11
Age: <35 years	168	34
>35 years	322	66
Marital status: Single	164	33
Married	281	57
Separated/Divorced/Widowed	45	9
Occupation: Unemployed	202	41
Daily wages	91	19
Self-employed	42	9
Salaried	130	26
Others	25	5
Income: <Rs 2500	68	14
Rs 2501–Rs 5000	216	44
>Rs 5000	179	36
Not applicable	27	6
Treatment Category: Category I	299	61
Category II	85	17
Category III	106	22
Rx phase: Intensive Phase	46	9
Continuation Phase	444	91

Of the 490 patients registered under RNTCP in 4 zones, 66% were males, 34% were below 35 years and 57% were married, Over half (58%) reported having a monthly family income of less than Rs 5000. 61% of the participants were treated under Category I, 17% under Category II and 22% under Category III.

### AUDIT Scoring Patterns

The total AUDIT score reflects the patient's level of risk related to alcohol. AUDIT scores are coded 0 to 4. When summed, score of 8, 16, and 20 or more indicate hazardous use, harmful use (alcohol abuse) and alcohol dependence respectively [14]. Four levels of risk are identified in alcohol use. Based on experience gained in a study of treatment matching with persons who had a wide range of alcohol problem severity, AUDIT scores were compared with diagnostic data reflecting low, medium and high degrees of alcohol dependence. It was found that AUDIT scores in the range of 8–15 represented a medium level of alcohol problems whereas scores of 16 and above represented a high level of alcohol problems. On the basis of experience gained from the use of the AUDIT in this and other research, it is suggested that the following interpretation be given to AUDIT scores: Scores between 8 and 15 are most appropriate for simple advice focused on the reduction of hazardous drinking. Scores between 16 and 19 suggest brief counseling and continued monitoring. AUDIT scores of 20 or above clearly warrant further diagnostic evaluation for alcohol dependence [14].

**Figure 2:**
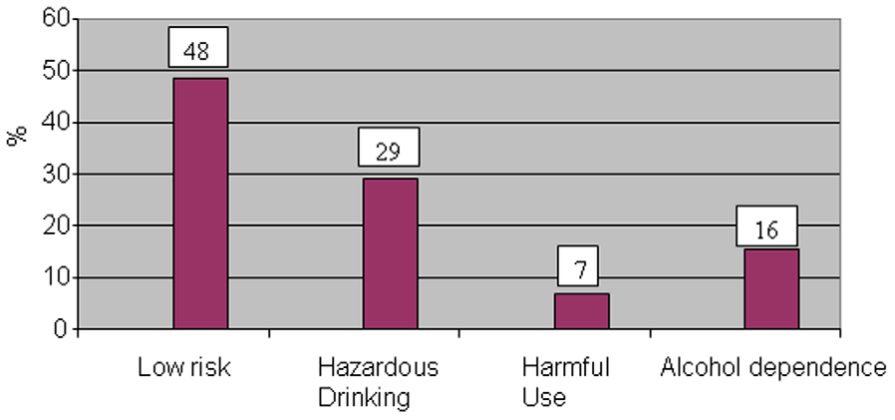
Different levels of alcohol consumption among TB patients (n = 141).

The chart (Figure 2) shows the distribution of TB patients with alcohol use Out of 490 TB patients, 29% (141) were found to consume alcohol. Of 141, 48% (68) were estimated to have low risk, 29% (41) hazardous drinking, 7% (10) harmful use and 16% (22) alcohol dependence.

### Association between Alcohol use and socio-demographic characteristics (Table 2)

**Table 2:** Association between Alcohol use and socio-demographic characteristics of TB patients (n = 490).

	TB patients without Alcohol use (n = 349)		TB patients with Alcohol use (n = 141)		P-value
	No.	%	No.	%	
Sex: (Sig: p<0.001)					
Male	181	52	141	100	0.000
Female	168	48	0		
Age:(Sig: p<0.001)					
<35 years	184	53	44	31	0.000
35 and above	165	47	97	69	
Marital Status: (Sig: p<0.05)					
Unmarried	130	37	34	24	
Married	182	52	99	70	0.012
Separated	10	3	2	2	
Divorcee	1	1	1	1	
Widow/Widower	26	7	5	3	
Education: (Sig: p<0.05)					
No formal education	56	16	19	14	0.013
School educated	246	70	115	82	
College educated	47	14	7	4	
Income (in Rs): (Sig: p<0.05)					
<2500	77	22	18	13	0.042
2501–5000	152	44	63	45	
>5000	120	34	60	42	
Treatment Category:					
(Sig: p<0.01)					
Category I	204	58	95	67	0.004
Category II	56	16	29	21	
Category III	89	26	17	12	

Overall 29% of the patients (141) were found to consume alcohol - exclusively being male patients. Of these 141 patients, 52% (73) had an AUDIT score >8. Aged over 35 years, a lower level of education, monthly income of <Rs 5,000, being separated or divorced, and a Category 2 (treatment failure/relapse) patient were found to be associated with an AUDIT score of greater than 8.

### Association between Alcohol use and treatment category

Out of 141 TB patients who consume alcohol, 112 patients were new patients and 29 were old patients. Among the new patients one half scored more than 8 by AUDIT and another half scored less than 8. But, among the old patients under Category II (29) that included treatment failure and relapse, 12 (41%) patients scored less than 8 by AUDIT and 17 (59%) scored more than 8. This data demonstrates that alcohol related problems are more prevalent among Category II.

### Treatment outcome and AUD (data not tabulated)

Treatment outcome of total 490 TB patients was compared with 141 TB patients who consume alcohol and 73 TB patients with AUDIT score >8 by checking the treatment registers at the respective health centres in the selected zones. There is not much difference among these 3 different groups regarding the cure rate. But, regarding the unfavorable responses of low risk group of <8 scores and 8 and above scores, failures were 43% in the former group against 57% in the later group. There were 2 deaths among TB patients with alcohol use. Similarly, default rate was 44% in low risk group whereas it was 56% among TB patients with.

## Discussion

The salient finding from this study reveals that almost a quarter of th TB patients who consumed alcohol could be classified as those with Alcohol use disorder (AUD). Furthermore an equal number were hazardous drinkers. This is in keeping with earlier studies that report that approximately half of those who drink alcohol show signs of dependence [22]–[24]. This group is vulnerable to risky behaviors and adverse health outcomes [25]. This is also worrisome as AUDs have been shown to be associated with worse treatment outcomes. [26]–[28]

With regard to the socio demographic variables influencing alcohol use lower level of education and low income were significant. This is paradoxical as alcohol users from poor households spend a large proportion of their income on alcohol thereby depleting resources that might otherwise be spent on health and education [29]. This may potentially lead to a vicious cycle between treatment outcomes and the patient's financial situation, as low income patients tended to have worse treatment outcomes and higher medical expenditure as result of treatment failure [30]. A study exploring the interlinkages between poverty, alcohol consumption and sexual health among men in India gives an indication of the close relationship between alcohol consumption and poverty [17]. In comparison to the non-poor, the extent of alcohol consumption was higher amongst the poorer sections of the community [31].

Other socio- demographic variables associated with alcohol use were aged over 35 years and being separated or divorced. It was also noted that re-treatment TB patients had higher AUDIT scores. This emphasizes the need for health providers to address such patients in order to identify alcohol associated problems in order that counseling services for this group could be strengthened.

It is also a matter of concern that treatment outcome was unsatisfactory for more than a tenth of those patients with AUD and hazardous drinking leading to chronic treatment default, treatment failure and death. Treatment failures were 43% in the low risk group with AUDIT score <8 against 57% in group of TB patients with AUDIT score >8. There were 2 deaths among 73 TB patients with AUD. Similarly, default rate was 44% in low risk group of <8 whereas it was 56% among the later group. This has been recorded by Greenfield et al [32] that alcohol disorders not only place individuals at increased risk for acquiring a number of diseases, but once people acquire a disease like TB, alcohol places them at higher risk for poor outcome and death.

Prevalence of alcohol use and AUD has been reported exclusively by males and none of the female patients reported alcohol use. This was similar to another community-based cross-sectional survey near Chennai in South India [9]. The reason for this could be that females are inhibited to disclose their alcohol use if any due to the social norms that stigmatize women drinkers and there is an extreme gender difference and prevalence among women which has consistently been estimated at <5% [22]. As per the estimates of National Survey Drug Abuse, 2004, there is an extreme gender difference and prevalence among women has consistently been estimated at <5%. In a series of studies of a probabilistic sample of 3600 households in Delhi it was reported that a prevalence of 7.0% and an incidence rate of 1.7% per annum of dependent drinking in men. No women were found in these categories. [33], [34] Similar findings in other states indicate that women are usually abstinent and that high percentages of men are also abstainers [35].

Alcohol use disorder and hazardous drinking among TB male patients is a matter of concern that needs to translate to an effective intervention program. In future a larger study will be conducted in which the intervention strategies for disorders related to alcohol use will be formulated on the basis of information collected.

### Conclusions

To conclude alcohol use disorder is a problem that needs to be addressed in the TB control programme. Effective measures and trained personnel to identify this disorder are an urgent and important requirement in major TB clinics. It is also important to identify the feasibility of alcohol intervention programs and to identify strategies based on patient's perceptions and needs.

### Limitations

This is a cross sectional study in an urban setting and therefore the findings of this study may not be generalizable outside of this setting. The study participants were TB patients attending government health facilities and our findings apply only to people attending government hospitals and not private hospitals. Thus our prevalence rates are likely to be higher than in the general population and overestimation may be considerable. The element of bias involved with self reported information on alcohol use also cannot be ruled out. This is because the concept of a standard measure of a drink amongst alcohol users is difficult to ascertain.
